# Time reversal and charge conjugation in an embedding quantum simulator

**DOI:** 10.1038/ncomms8917

**Published:** 2015-08-04

**Authors:** Xiang Zhang, Yangchao Shen, Junhua Zhang, Jorge Casanova, Lucas Lamata, Enrique Solano, Man-Hong Yung, Jing-Ning Zhang, Kihwan Kim

**Affiliations:** 1 Center for Quantum Information, Institute for Interdisciplinary Information Sciences, Tsinghua University, Beijing 100084, China; 2 Institut für Theoretische Physik, Universität Ulm, Albert-Einstein-Allee 11, Ulm D-89069, Germany; 3 Department of Physical Chemistry, University of the Basque Country UPV/EHU, Apartado 644, Bilbao E-48080, Spain; 4 IKERBASQUE, Basque Foundation for Science, Maria Diaz de Haro 3, Bilbao 48013, Spain

## Abstract

A quantum simulator is an important device that may soon outperform current classical computations. A basic arithmetic operation, the complex conjugate, however, is considered to be impossible to be implemented in such a quantum system due to the linear character of quantum mechanics. Here, we present the experimental quantum simulation of such an unphysical operation beyond the regime of unitary and dissipative evolutions through the embedding of a quantum dynamics in the electronic multilevels of a ^171^Yb^+^ ion. We perform time reversal and charge conjugation, which are paradigmatic examples of antiunitary symmetry operators, in the evolution of a Majorana equation without the tomographic knowledge of the evolving state. Thus, these operations can be applied regardless of the system size. Our approach offers the possibility to add unphysical operations to the toolbox of quantum simulation, and provides a route to efficiently compute otherwise intractable quantities, such as entanglement monotones.

## Abstract (web summary)

Quantum simulation has the potential to enable experimentally studying problems which are not directly tractable in a laboratory or computationally. Here, the authors simulate the Majorana equation with a ytterbium ion, which requires them to simulate antiunitary and therefore unphysical operations.

## Graphical abstract

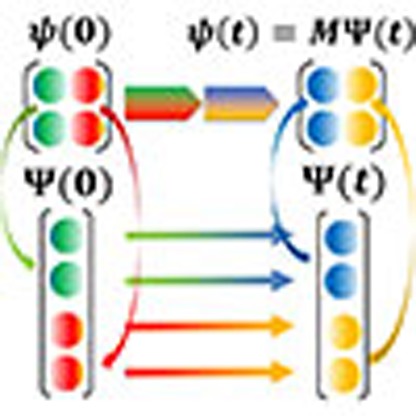

Quantum computers or quantum simulators are important quantum devices that may enable us to experimentally address intriguing phenomena that are not directly tractable in the laboratory^1^ or may outperform current classical computations for analysing complex quantum systems^2^,^3^. In recent years, various physical platforms such as neutral atoms^4^, ions^5^, photons^6^ and superconducting circuits^7^ have been fruitfully developed for the purpose of quantum simulation. However, they are not yet able to perform some basic arithmetic calculations such as the complex conjugate, which changes the sign of the imaginary part of the coefficients of the state on a certain basis. Although we are used to computing the transformation with classical resources for useful scientific calculations, operations involving the complex conjugate require an antiunitary process, which is impossible to be implemented in a quantum system. Moreover, the complex conjugate is not scalable in classical calculation, since it requires full knowledge of the quantum state, and the number of measurements grows exponentially with the size of the system.

The complex conjugate is deeply inherent to the important concepts of discrete symmetries. Wigner^8^ proved that any symmetry operation acts as a unitary or antiunitary transformation in the Hilbert space, while an antiunitary transformation can always be decomposed into a unitary transformation together with the complex conjugate. The study of symmetries has profoundly shaped our comprehension of physical laws in the quantum field theory, which unifies quantum mechanics and special relativity. Charge conjugation and time reversal are paradigmatic examples of antiunitary discrete symmetry operations^9^,^10^. The charge conjugation, together with the parity symmetry, is not conserved in the weak interaction^11^,^12^, just as the time-reversal symmetry. The discovery of the violation of these symmetries has been a decisive breakthrough of the quantum field theory, leading to the standard model. Recently, several important algorithms for the simulation of relativistic quantum mechanics and quantum field theory have been discovered^13^,^14^,^15^,^16^,^17^,^18^. So far, however, quantum simulators of unitary and dissipative processes, the only physically allowed dynamics, have been realized^19^,^20^,^21^.

Here, we perform the quantum simulation of the complex conjugate and these symmetry operations in our multilevel ^171^Yb^+^ ion system through the use of the concept of embedding quantum simulator (EQS)^22^,^23^,^24^ beyond the boundary of the unitary operations. Our demonstration is scalable, where we can apply the time-reversal or charge-conjugation operations in systems of any size, since they do not require the tomographical knowledge of the state. The essence of the EQS is based on the finding that antiunitary operations can be implemented in a physical system by doubling the associated Hilbert space^22^. The scheme of the EQS enables us to efficiently compute entanglement monotones^23^ or multi-time correlation functions^24^,^25^. The reconstruction of these quantities would otherwise require a number of measurements that grows exponentially with the system size. We comment that the measurement of such quantities can be considered as an intractable task even for medium-size systems composed by, for example, only a dozen of qubits, whereas the EQS scheme provides the solution at the expense of one additional qubit to double the Hilbert space.

## Results

### Majorana dynamics

We first simulate the Majorana dynamics to show the ‘unphysical' capability of the EQS before implementing antiunitary symmetry operations. The Majorana equation^26^, one of the representative relativistic equations,


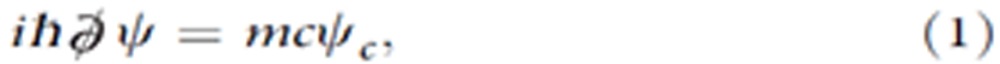


where the Feynman slash notation 
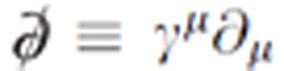
 with 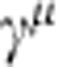 being the Dirac matrices^27^, describes the dynamics of a non-Hamiltonian system. Note that the spinor 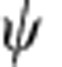 and its charge conjugation 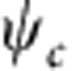 are present simultaneously in equation (1). Majorana envisioned that equation (1) together with the Majorana condition 
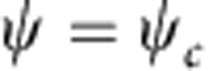
 would be the fundamental equation describing neutrinos^26^, which exhibit the novel phenomenon as ‘neutrino oscillation'^28^. Besides, the Majorana equation (1) has its own theoretical importance in exploring physics beyond the standard model. Moreover, the utility of the relativistic equations is not limited to relativistic quantum mechanics and the quantum field theory. For example, electrons propagating through graphene are described by the (2+1)-dimensional Dirac equation^29^, and the symmetry breaking induced by tachyon condensation is described by a (1+1)-dimensional Dirac-like equation with imaginary mass^30^, a non-Hamiltonian system. Recently, a quantum simulation of the Majorana dynamics was performed in a photonic quantum platform, by decomposing its evolution in two Dirac equations^31^,^32^. Through the quantum simulation of the inherently unphysical Majorana equation, we demonstrate various unique features, such as violation of charge and momentum conservation, broken orthogonality and nontrivial effect of the state's global phase.

### Embedding quantum simulator

**Figure 1:**
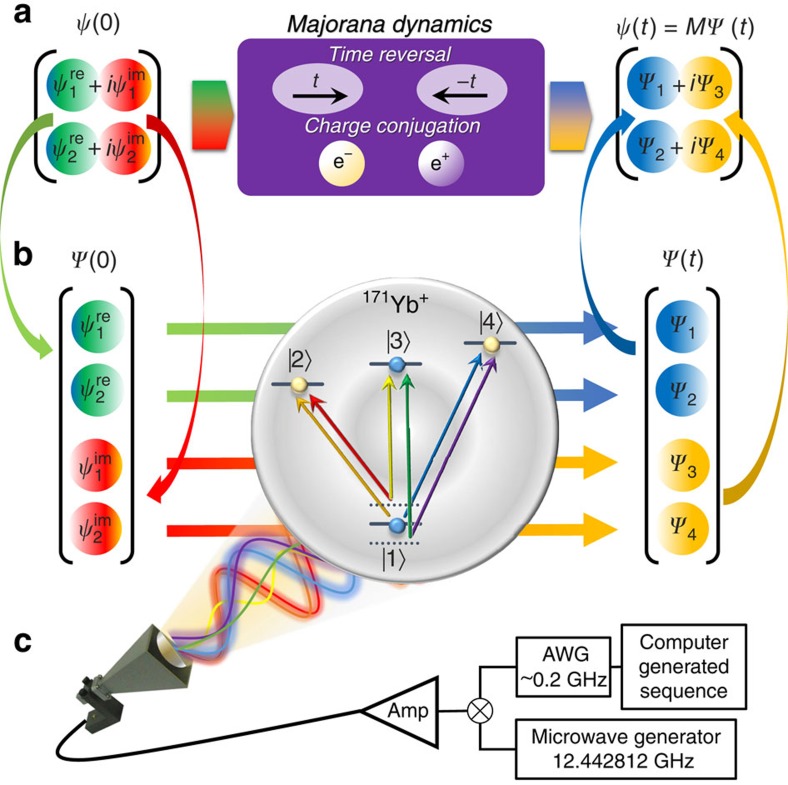
Schematic of the embedding quantum simulation (EQS) (**a**) The ‘unphysical' process in the original Hilbert space, where the evolution of the two-component Majorana spinor is considered. We also implemented the unphysical processes, such as the time-reversal and charge-conjugation operations during the Majorana dynamics, which are forbidden by the laws of quantum mechanics. (**b**) The unphysical processes can be implemented in physical systems through the embedding scheme, which maps the original Hilbert space to the enlarged Hilbert space. After the physical process, the final results are remapped to the original space. The embedding quantum simulator is built in a single ^171^Yb^+^ ion trapped in a linear Paul trap, where the enlarged space is encoded in the ground-state manifold of the ion. (**c**) The physical unitary operations in the enlarged Hilbert space are implemented by applying microwaves with six frequencies, which are generated by mixing the monochromatic microwave (12.442812 GHz) with the signals from computer-programmed arbitrary wave form generator.

The essential idea of an EQS is the mapping from the original Hilbert space 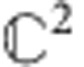 to the enlarged one 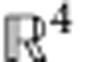 for spinors in 1+1 space-time dimension, 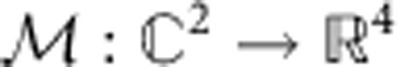. In the position basis, as shown in Fig. 1, the EQS mapping is defined as


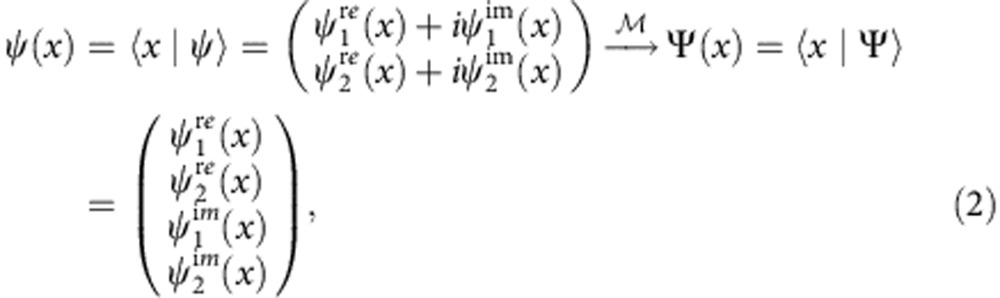


where 
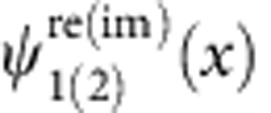
 are real functions satisfying the overall normalization condition 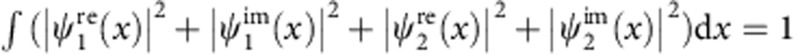. Inversely, as depicted in Fig. 1, the original spinor is retrieved through a matrix multiplication after evolving in the EQS for certain duration,


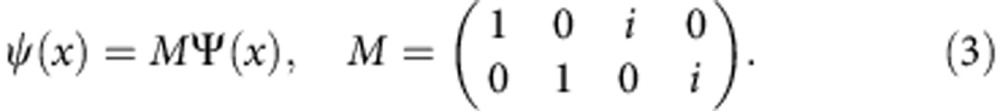


Through the EQS mapping (equation (3)), the complex conjugate operation, 
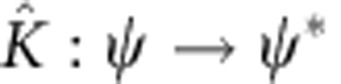
, is represented by a unitary operator 
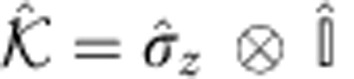
 in the enlarged Hilbert space, which can be implemented directly in a quantum system (see Methods and Supplementary Note 1).

With a certain choice of the Dirac matrices in the (1+1) dimension, 
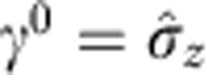
 and 
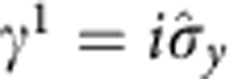
, the charge-conjugate spinor is properly defined as 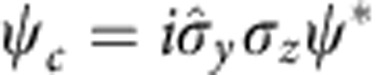. The Majorana equation in 1+1 dimensions,


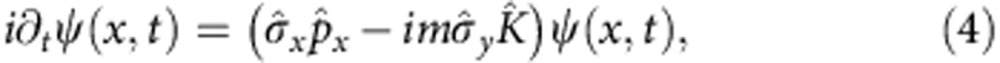


inherently contains the complex conjugate operator 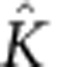, which makes the Majorana dynamics prohibited by nature. For simplicity, we introduce a set of dimensionless units, that is, *mc*^2^ for the energy, *mc* for the momentum and 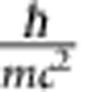 for the time.

In the enlarged Hilbert space, the original non-Hamiltonian system is mapped to a Hamiltonian one governed by an effective Hamiltonian,


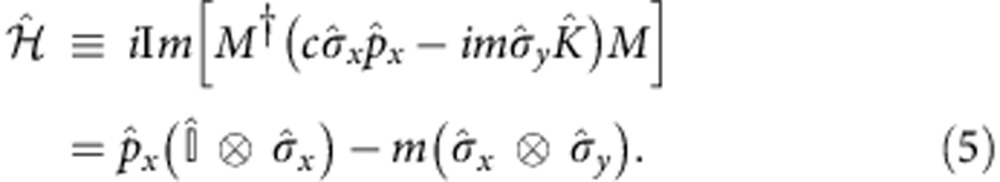


Note that the equation of motion in the enlarged space, 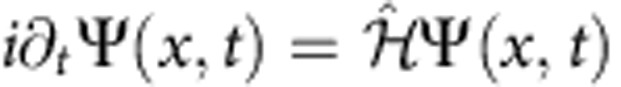, keeps 
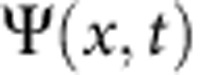
 evolving inside 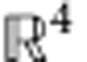. Because the effective Hamiltonian (equation (5)) does not contain a position operator, we perform the experimental implementation in momentum representation, where the dynamics of the Fourier-transformed spinor 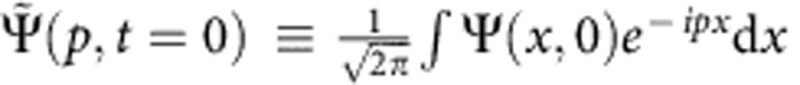 is governed by a simpler Hamiltonian 
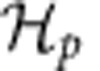
 obtained by substituting the momentum operator with its eigenvalue in equation (5) (see Supplementary Note 2).

Along the same line, some discrete symmetry operations, that is, the time reversal 
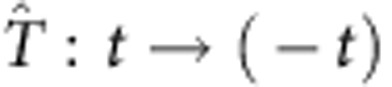
 and the charge conjugation 
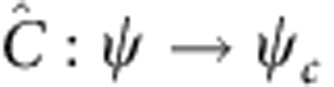
 take form of unitary two-qubit gate operations in the enlarged Hilbert space: 
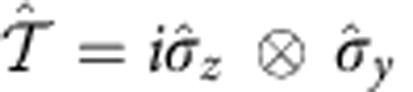
 and 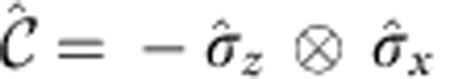, respectively.

### Experimental set-up

The EQS is built in an ion-trap system, which is a leading platform for quantum simulation^5^. The system consists of a single ^171^Yb^+^ ion confined in a linear Paul trap^33^, subjected to multifrequency microwaves. As shown in Fig. 1, the four internal states of the ground-state manifold ^2^*S*_1/2_ are encoded as |*F*=0, *m*_*F*_=0〉≡|1〉 and |*F*=0, *m*_*F*_=−1, 0, 1〉≡|*m*_*F*_+3〉, |1〉 and {|2〉, |3〉, |4〉} are separated by the hyperfine splitting 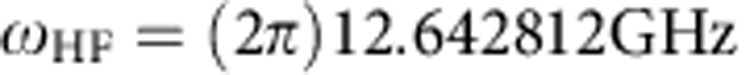, and a uniform static magnetic field *B*=9.694 G is applied to define the quantization axis and causes Zeeman splitting 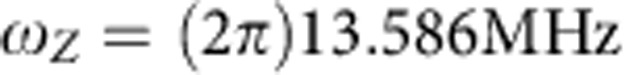 among the upper states. As shown in the Fig. 1, the couplings between |1〉 and the upper states can be directly driven by microwave with frequencies as 
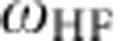
 and 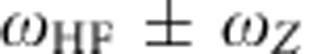, respectively. The couplings among the equally spaced upper states, that is, |2〉, |3〉 and |4〉, are implemented by the stimulated Raman process of microwaves (see Methods). On the basis of the multi-fold microwave technique, we achieve ultimate controllability over the Hilbert space spanned by all of the four internal states. In other words, we construct a ququad, an elementary unit of quantum information processing consisting of four basis states. In principle, large-scale EQS can always be constructed by substituting one of the qubits in an array by a ququad, and the requisite microwave techniques involved in the control of the ququad have been developed in this work.

With the ability to perform any single-ququad operation, we implement the effective Hamiltonian equation (5) in the momentum space,


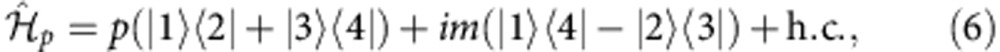


on top of the EQS.

### Experimental procedure

The experimental procedure is as follows. First, we map an initial Majorana spinor 
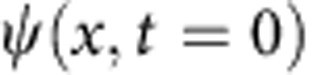
 to a real bispinor 
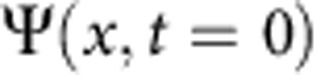
 in the enlarged space. The momentum representation of the bispinor 
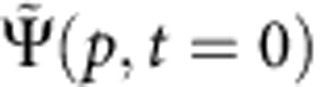
 evolves according to the enlarged space Hamiltonian 
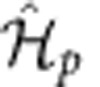
. After encoding the initial condition 
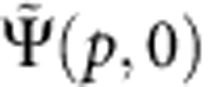
 into the EQS, we implement 
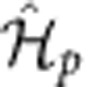
 for a certain duration to simulate the Majorana dynamics. Then we perform quantum-state tomography (see Supplementary Note 3) to obtain the enlarged space density matrix 
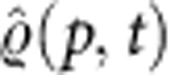
, which can be mapped to the original space density matrix 
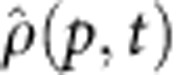
. The average value of a diagonal operator *A*_*d*_ in the momentum space can be directly obtained via integration over the momentum, 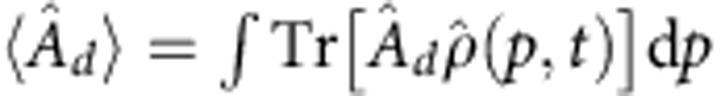. To obtain the average value of an off-diagonal operator in the momentum space, for example, the average position of the Majorana particle, we change the four-component equation of motion in the enlarged Hilbert space into a pair of decoupled two-component equations by diagonalizing the first qubit (see Supplementary Note 4). By coherently evolving a couple of two-dimensional equations with different momenta, we obtain the phase information between different momentum components. We repeat each measurement 1,000 times to get the expectation value. The statistical errors, which are mainly due to quantum projection, are estimated by the s.d. of mean value.

### Majorana dynamics

**Figure 2:**
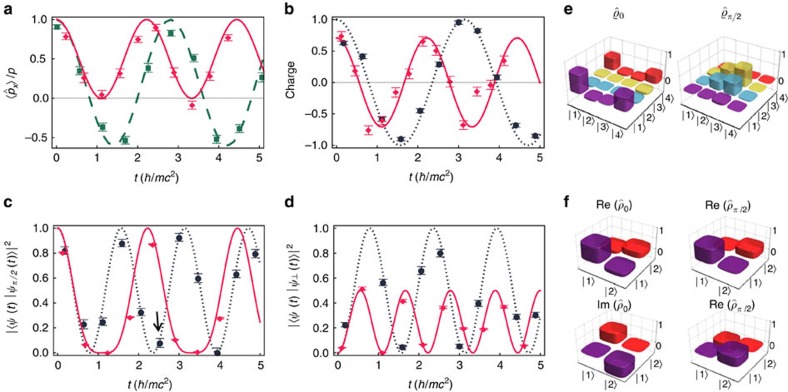
Majorana dynamics. (**a**) *Zitterbewegung* in momentum space during the evolution of a single Majorana particle. (**b**) Violation of charge conservation in the Majorana dynamics. The average values of the physical observables in **a** and **b** are measurement results of the Majorana spinor 
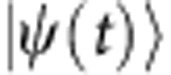
 evolving from the initial state 
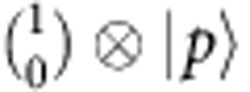
. (**c**) Nonconserved fidelity caused by an initial global phase. The Majorana spinors 
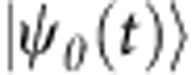
 evolve from the initial states 
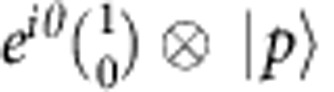
 with *θ*=*π*/2. (**d**) Nonconserved orthogonality for initially perpendicular Majorana spinors. The Majorana spinors 
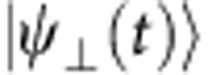
 evolve from initial states 
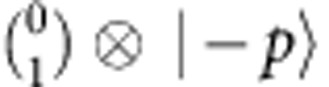
. We choose the momenta of the initial plane-wave states as *P*=0 (black dots and dotted line), 0.5 (green squares and dashed line) and 1 (red diamonds and solid line), where Majorana mass *m*=1. The curves are from theoretical simulation. (**e**) Density matrices in the enlarged space obtained by quantum-state tomography, related to the data point marked by the black arrow in **c**. (**f**) Reconstructed density matrices in the original space. The value of each observable for the state tomography in the enlarged space is measured by averaging the experimental results after 1,000 times repetition. We estimate the error bars in **a**–**d** by using the standard error propagation method of the standard deviation (1***σ***) of each observable in the tomography mainly from the quantum projection noise (see Supplementary Note 3).

Figure 2 shows our experimental results of the Majorana dynamics, where the initial spinors are chosen to be plane-wave states with 
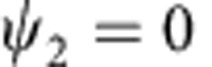
, that is, 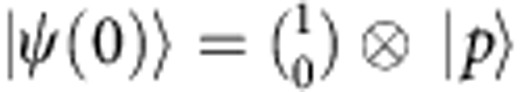. Figure 2a shows the momentum-space *Zitterbewegung* for a Majorana particle. Due to the existence of the complex conjugate operator in the Majorana equation, the momentum, which is conserved for free Dirac particles, is no longer a conserved quantity in the Majorana dynamics. Because the violation of momentum conservation is originated by the Majorana mass term, the amplitude of the oscillation is inversely proportional to the magnitude of the momentum of the initial state. Meanwhile, the frequency of the oscillation is determined by the relativistic dispersion relation 
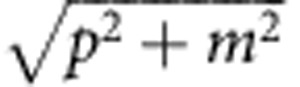
, so the initial plane wave with larger momentum will oscillate faster. As shown in Fig. 2b, the Majorana dynamics also violates charge conservation, which may lead to physics beyond the standard model^34^. In the rest frame, the charge operator measures the difference between the populations of the internal states, which is equivalent to the 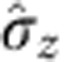 operator^35^. For the non-zero momentum case, the particle and antiparticle basis is obtained by diagonalizing the corresponding Dirac equation with the same momentum, and the charge of a Majorana spinor is defined as the difference between the populations of the particle and antiparticle components (see Supplementary Note 5). For the same reason, the amplitude and frequency of the charge oscillation exhibits similar momentum dependence as that of the momentum-space *Zitterbewegung*.

Besides the above physical consequences, the dynamics governed by Majorana equation also shows unphysical phenomena. For example, the fidelity 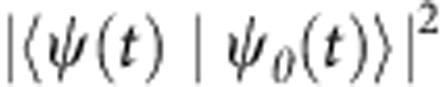, where 
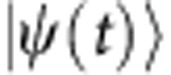
 and 
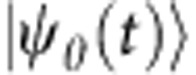
 are two Majorana spinors that evolve from initial states differing only in a global phase, 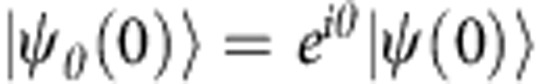, will not always be unity as shown in Fig. 2c. In other words, a Majorana spinor does not have the freedom to choose an arbitrary global phase. The reason for this surprising effect is the existence of the complex conjugate 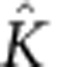 in the Majorana equation in equation (4). This effect can be more explicitly shown in the mapping 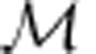 in equation (2), that is, the global phase actually changes the initial four-component spinor in the enlarged Hilbert space. Figure 2e,f shows an example of the experimental results of the density matrices in the enlarged and original Hilbert spaces, which are indeed different from each other. In Fig. 2d, we experimentally observe the non-conservation of the orthogonality defined as 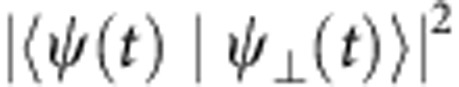, with 
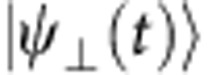
 being the Majorana spinor evolved from an orthogonal initial state 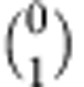. During the evolution, the initial Majorana spinor will be coupled to 
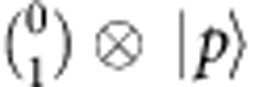
 through the Hermitian relativistic kinetic term 
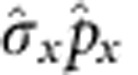
, and 
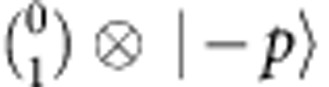
 through the non-Hermitian Majorana mass term 
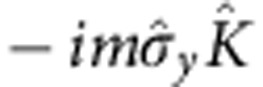
. The orthogonality 
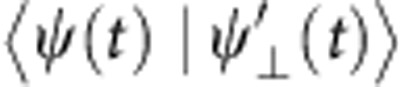
, where 
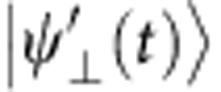
 is the Majorana spinor that evolves from the initial state 
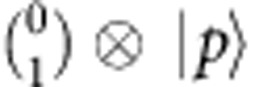
, is always zero. This clearly indicates that the non-conservation of the orthogonality 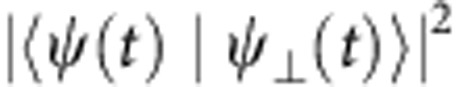 stems from the non-Hermitian part of the Majorana Hamiltonian. As a result, given the same Majorana mass, we understand that the amplitude of the orthogonality oscillation is inversely proportional to the initial momentum.

### Symmetry operations

**Figure 3:**
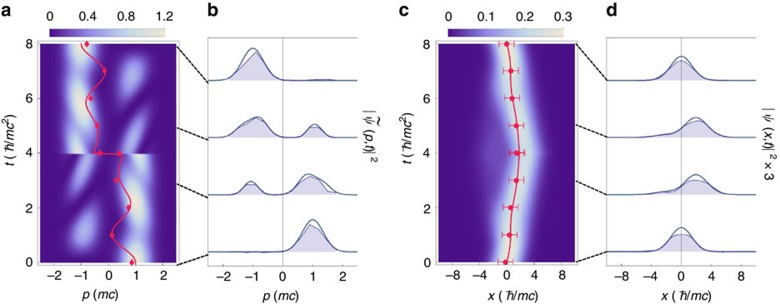
Time-reversal operation. The time-reversal operation is performed at the mid-point (*t*=4) of the evolution of a Majorana particle. The initial state is a moving Gaussian wave packet 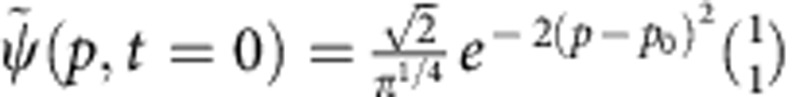 in momentum space and 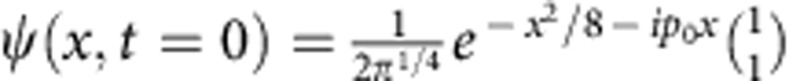 in position space, respectively, with initial average momentum *P*_0_=1. (**a**) Time-dependent probability distribution in the momentum space. The solid curve represents the theoretical results of the average momentum 
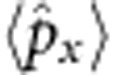
, whereas the red dots with error bars are experimental results. The same conventions are used for **c**, and **a** and **c** of Fig. 4. Right after the time-reversal operation at *t*=4, the momentum *p* of the Majorana particle is reversed to −*p*, which makes the discontinuity at the evolution of the picture. It is clearly shown that the momentum-space *Zitterbewegung* is revived after the time-reversal operation. (**b**) Probability distributions in the momentum space at various times *t*=0, 3, 5 and 8 from bottom to top. The solid curves are obtained from theoretical calculation whereas the shades are from experiment. The same conventions are used for **d**, and **b** and **d** of Fig. 4. (**c**) Time-dependent probability distribution in the position space. The average position is not affected right after the time-reversal operation, but the trajectory is reversed. (**d**) The same as **b** in the position space. The error bars in **a** and **c** are estimated by the standard error propagation method of the measured observables with mainly the standard deviation (1***σ***) of quantum projection noise (see Supplementary Note 3).

**Figure 4:**
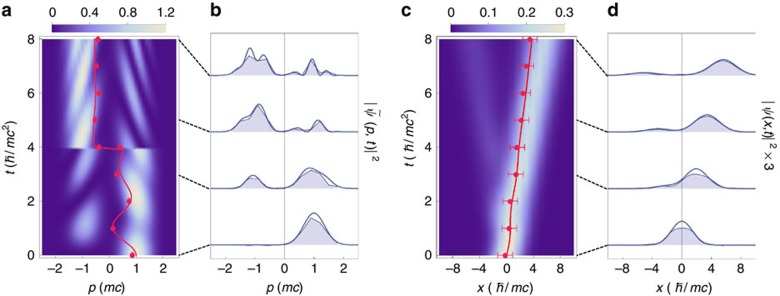
Charge-conjugation operation. For the same initial state in Fig. 3, the charge-conjugation operation is performed at the mid-point (*t*=4). (**a**) Time-dependent probability distribution in the momentum space. Similar to the time-reversal operation, right after the charge-conjugation operation at *t*=4, the momentum *p* is reversed to −*p*, which makes the discontinuity at the evolution of the picture. However, the momentum-space *Zitterbewegung* fades away, which is clearly different from the result of the time-reversal operation. (**b**) Probability distributions in the momentum space at various times *t*=0, 3, 5 and 8 from bottom to top. (**c**) Time-dependent probability distribution in the position space. Although the charge-conjugation has changed the internal components of the Majorana spinor (see Supplementary Fig. 1), the trajectory of the Majorana particle is not affected by the charge-conjugation operation. (**d**) The same as **b** in the real space. The corresponding data points are marked by blue dashed lines. The error bars in **a** and **c** come from the same method to those in Fig. 3.

Other than the plane waves, we also implement Majorana dynamics with realistic initial wave packets in our EQS. For example, the initial states for the Majorana dynamics in Figs 3 and 4 are moving Gaussian states with momentum distributions centred around *P*_0_=1 with an internal state 
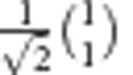
. The first part of the time axis (0≤*t*<4) in Figs 3 and 4 represent the Majorana dynamics of a moving wave packet, where we observe damping oscillation in the momentum space and *Zitterbewegung* in the position space. The reason of the damping in the momentum space is that a Gaussian wave packet has distribution over many different momentum components, and each momentum component oscillates with different frequency. To our surprise, although the average momentum of a Majorana particle behaves quite different from that of a Dirac particle, there is no visible difference in the behaviours of the average position as well as the probability distribution in position space. This is because a Majorana particle oscillates between the particle and antiparticle components with inverse momentum, but the positions as well as the velocities of the particle and antiparticle are exactly the same^9^.

During the evolution of the Majorana equation, we implement the antiunitary time-reversal and charge-conjugation operations. Figure 3 shows our experimental results of the time-reversal operation during the Majorana time evolution. As shown in Fig. 3a, right after the time-reversal operation, the momentum as well as the velocity changes sign. As a result, the direction of the wave packet is reversed as shown in Fig. 3c. The damped average momentum as well as the position centre of the wave packet is revived, which clearly shows that time is indeed reversed. Figure 4 demonstrates the experimental implementation of the charge-conjugation operation. The latter interchanges the particle and antiparticle components, which are defined from the corresponding Dirac equation with the same momentum as discussed in Fig. 2b. By definition, the particle and corresponding antiparticle have opposite momentum but the same velocity. As a result, right after the charge-conjugation operation, the average momentum is reversed but not the velocity. Therefore, the trajectory in position space remains intact, which is different from the time-reversal operation.

## Discussion

The demonstrated embedding scheme would potentially reduce the computational complexity of ordinary quantum simulations in the sense that it eliminates the requirements for tomographic information. By enlarging the EQS, the demonstrated symmetry operations, can be potentially scaled up to many-particle systems in higher space-time dimensions, in which the conventional quantum-state tomography is theoretically impossible. The EQS for multipartite systems only requires doubling the original Hilbert space dimension, which can be achieved by replacing a single qubit in an array of coupled qubits by a ququad (quantum four-level system). The proposed embedding scheme for the implementation of time reversal and charge-conjugation operations may be extended to parity symmetry operations^24^. This enhanced toolbox for quantum simulators will be valuable for studying conservation laws and improving the computational capabilities of current quantum platforms.

## Methods

### Example of EQS mapping

In the following, we use a plane-wave initial state 
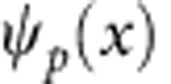
 as an example of the encoding of states in the enlarged Hilbert space,


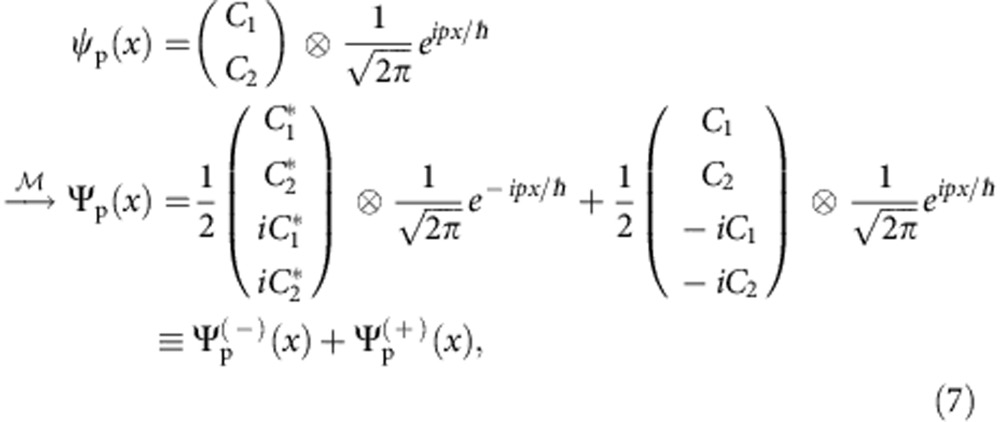


where 
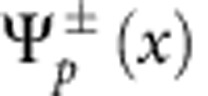
 corresponds to plane-wave states (unnormalized) with momentum ±*p*. Here we want to emphasize two points: (i) although 
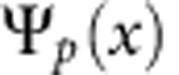
 is real, the components 
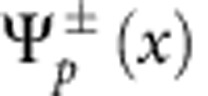
 are usually composed of complex functions; (ii) there are always +*p* and −*p* components in the enlarged space to guarantee 
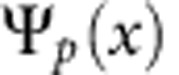
 is real.

### Stimulated Raman couplings

We implement a microwave Raman scheme for the transitions between |2〉 to |3〉 and |3〉 to |4〉. The strengths of effective Raman couplings are given by 
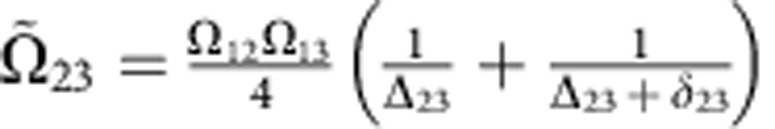
 and 
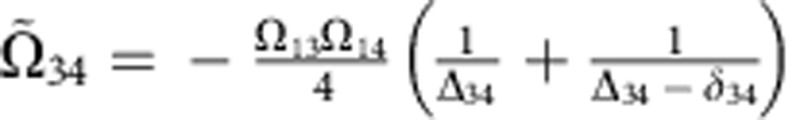
, where 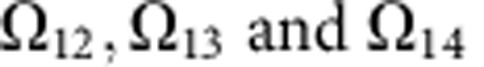 are Rabi frequencies between |1〉 to |2〉, |1〉 to |3〉, and |1〉 to |4〉, respectively, 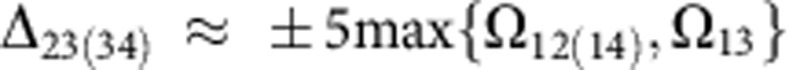 are the detuning from |1〉 to |2〉(|4〉) and *δ*_23(34)_ are frequency shifts for the compensation of AC-Stark effect between |1〉 to |3〉. The strengths of the Raman transitions are balanced to the direct transitions, which have around (2*π*)3 kHz. The cross talks between two transitions 
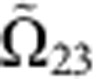
 and 
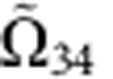
 are negligible because 
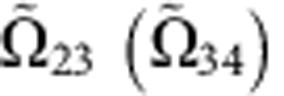
 is produced by the combination of *σ*_−_(*σ*_+_) and *π* polarizations of microwave, which is impossible to couple to |3〉↔|4〉 transition (|2〉↔|3〉). The AC-Stark shifts from all the microwave transitions are carefully compensated by properly adjusting the microwave frequencies (see Supplementary Note 6).

## Additional information

**How to cite this article:** Zhang, X. *et al.* Time reversal and charge conjugation in an embedding quantum simulator. *Nat. Commun.* 6:7917 doi: 10.1038/ncomms8917 (2015).

## Supplementary Material

Supplementary Information Supplementary Figure 1, Supplementary Note 1-6 and Supplementary References
