# Supplementary material for: Time reversal and charge conjugation in an embedding quantum simulator
Source: Nat Commun. 2015 Aug 4;6:7917. doi: 10.1038/ncomms8917 (PMC4532877; doi:10.1038/ncomms8917)
Supplement: Supplementary Information — Supplementary Figure 1, Supplementary Note 1-6 and Supplementary References [file ncomms8917-s1.pdf]

Supplementary Figures

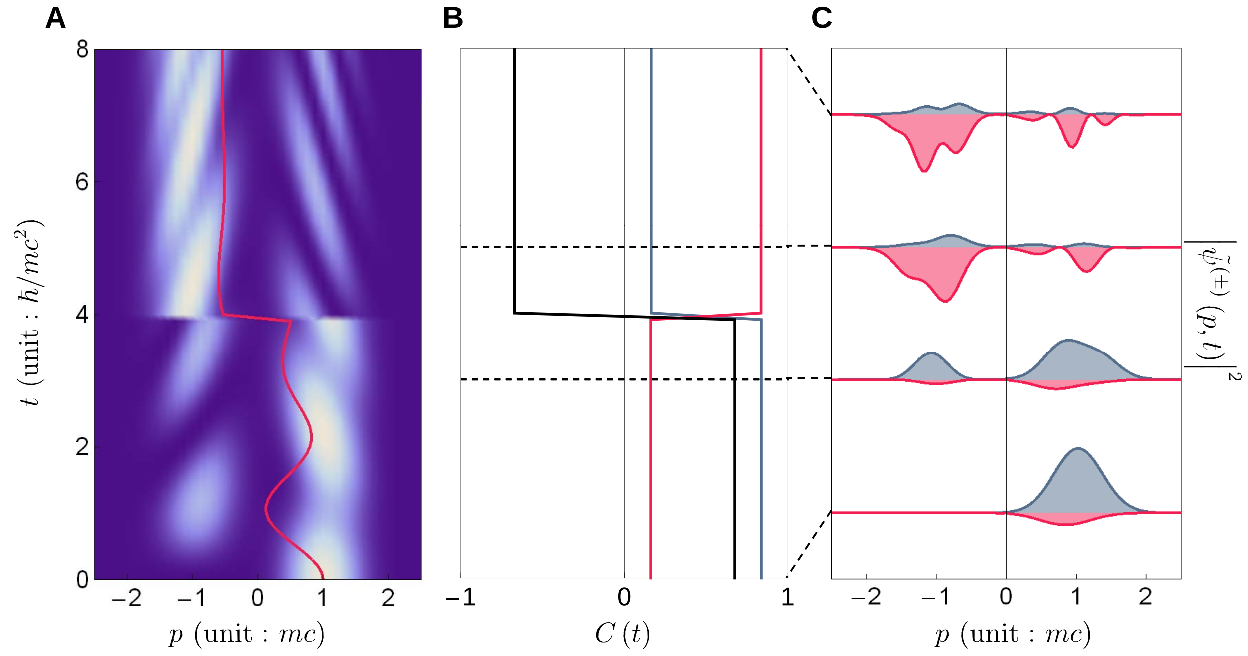

Supplementary Figure 1: **Charge conjugation on top of the Majorana dynamics. a.** Time-dependent density distributions in momentum space. We implement the charge conjugation at the intermediate time $t = 4$. The solid line represents the average value of the momentum. **b.** Time-dependent charge (black) as well as populations of the particle (blue) and antiparticle (red) components. **c.** Momentum distributions of the particle (blue) and antiparticle (red) components at different times $t = 0$, 3, 5, and 8. The curves are displaced along the vertical axis for better visualization.

Supplementary Note 1: Mapping Between Original and Enlarged Spaces

In order to implement unphysical operations, such as the time reversal and charge conjugation, in our prototype of the embedding quantum simulator, we first consider the mapping $\mathcal{M}$ which transforms the state vector in the original n-dimensional complex Hilbert space

$\mathbb{C}^n$ onto an enlarged $2n$-dimensional real Hilbert space $\mathbb{R}^{2n}$,

$$\psi(x)=\begin{pmatrix}\psi_1(x)\\ \vdots\\ \psi_n(x)\end{pmatrix}\longrightarrow\mathcal{M}\Psi(x)=\frac{1}{2}\begin{pmatrix}\psi_1(x)+\psi_1^*(x)\\ \vdots\\ \psi_n(x)+\psi_n^*(x)\\ i\left(\psi_1^*(x)-\psi_1(x)\right)\\ \vdots\\ i\left(\psi_n^*(x)-\psi_n(x)\right)\end{pmatrix}\equiv\begin{pmatrix}\psi_1^{\mathrm{re}}(x)\\ \vdots\\ \psi_n^{\mathrm{re}}(x)\\ \psi_1^{\mathrm{im}}(x)\\ \vdots\\ \psi_n^{\mathrm{im}}(x)\end{pmatrix}. \tag{1}$$

In the $1+1$ dimension case, we consider the specific mapping $\mathcal{M}:\mathbb{C}^2\rightarrow\mathbb{R}^4$. In the following, we use a plane-wave initial state $\psi_p(x)$ as an example of the encoding of states in the enlarged Hilbert space,

$$\begin{aligned}\psi_p(x) &= \begin{pmatrix}C_1\\ C_2\end{pmatrix}\otimes\frac{1}{\sqrt{2\pi}}e^{ipx/\hbar} \qquad (2)\\ \longrightarrow\mathcal{M}\Psi_p(x) &= \frac{1}{2}\begin{pmatrix}C_1^*\\ C_2^*\\ iC_1^*\\ iC_2^*\end{pmatrix}\otimes\frac{1}{\sqrt{2\pi}}e^{-ipx/\hbar}+\frac{1}{2}\begin{pmatrix}C_1\\ C_2\\ -iC_1\\ -iC_2\end{pmatrix}\otimes\frac{1}{\sqrt{2\pi}}e^{ipx/\hbar}\\ &\equiv \Psi_p^{(-)}(x)+\Psi_p^{(+)}(x),\end{aligned}$$

where $\Psi_p^{\pm}(x)$ corresponds to plane-wave states (unnormalized) with momentum $\pm p$. Here we want to emphasize two points: (i) although $\Psi_p(x)$ is real, the components $\Psi_p^{\pm}(x)$ are usually composed of complex functions; (ii) there are always $+p$ and $-p$ components in the enlarged space to guarantee $\Psi_p(x)$ is real.

Supplementary Note 2: Quasi-quantum Treatment of the Momentum Operator

The $1+1$ Majorana equation for a two-component complex spinor $\psi(x)=\begin{pmatrix}\psi_1(x)\\ \psi_2(x)\end{pmatrix}$ is mapped onto a $3+1$ Dirac equation for a four-component real bispinor $\Psi(x)=\begin{pmatrix}\psi_1^{\mathrm{re}}(x)\\ \psi_2^{\mathrm{re}}(x)\\ \psi_1^{\mathrm{im}}(x)\\ \psi_2^{\mathrm{im}}(x)\end{pmatrix}$ in the enlarged space, which takes the following form,

$$i\hbar\partial_t\Psi(x,t)=\hat{\mathcal{H}}\Psi(x,t)=\left[c\hat{p}_x\left(\hat{\mathbb{I}}\otimes\hat{\sigma}_x\right)-mc^2\left(\hat{\sigma}_x\otimes\hat{\sigma}_y\right)\right]\Psi(x,t), \tag{3}$$

where $\hat{p}_x=-i\hbar\partial_x$ is the momentum operator.

In the momentum space, the bispinor $\widetilde{\Psi}(p,t)$ is obtained via the Fourier transformation,

$$\widetilde{\Psi}(p,t) = \frac{1}{\sqrt{2\pi}} \int \Psi(x,t)\, e^{-ipx/\hbar} dx, \tag{4}$$

and the equation of motion becomes

$$i\hbar\partial_t \widetilde{\Psi}(p,t) = \hat{\mathcal{H}}_p \widetilde{\Psi}(p,t) = \left[cp\left(\hat{\mathbb{I}} \otimes \hat{\sigma}_x\right) - mc^2\left(\hat{\sigma}_x \otimes \hat{\sigma}_y\right)\right] \widetilde{\Psi}(p,t), \tag{5}$$

where the momentum operator $\hat{p}_x$ is substituted by its eigenvalue p. It is clear that the dynamics governed by $\hat{\mathcal{H}}_p$ is ready to be implemented in a quantum four-level system.

Supplementary Note 3: Quantum State Tomography

We measure a state's density matrix by performing qudit state tomography[3]. The density matrix of a state in the 4-level system can be written as

$$\hat{\rho}_4 = \frac{1}{2}\sum_{j=1}^{15} r_j \hat{\lambda}_j + \frac{1}{4}\hat{I}_4,$$

where $\hat{\lambda}_j$ are the SU(4) generators, which are referred as four dimensional generalized Gell-Mann matrices[4]. They have following properties:

$$\mathrm{Tr}(\hat{\lambda}_j) = 0, \mathrm{Tr}(\hat{\lambda}_j \hat{\lambda}_k) = 2\delta_{jk}.$$

Thus we can have

$$r_j = \mathrm{Tr}(\hat{\rho}_4 \hat{\lambda}_j) = \langle \hat{\lambda}_j \rangle,$$

and the density matrix $\hat{\rho}_4$ can be reconstructed by measure all expectation values $\langle \hat{\lambda}_j \rangle$.

In our trapped-ion system, the florescence detecting method can only directly measure population on state $|1\rangle$. But it can be easily extended to measure the population on other state $|n\rangle$ by state flipping with a π pulse resonant at $|1\rangle \leftrightarrow |n\rangle$ transition. For a diagonal operator $\hat{A} = \mathrm{diag}\{a_1, a_2, a_3, a_4\}$, its expectation value can be calculated by

$$\langle \hat{A} \rangle = \mathrm{Tr}(\hat{\rho}_4 \hat{A}) = \sum_{j=1}^{4} a_j \rho_{jj},$$

where ρ_{jj} is the population on state $|j\rangle$. For a off-diagonal operator $\hat{B}$, we can diagonalize $\hat{B}$ to $\hat{B}_d = \hat{U}\hat{B}\hat{U}^\dagger$. The expectation value of $\hat{B}$ on state ψ can be written as

$$\langle \hat{B} \rangle = \langle \psi | \hat{B} \psi \rangle = \langle \hat{U}\psi | \hat{B}_d | \hat{U}\psi \rangle,$$

which is just the expectation value of diagonal operator $\hat{B}_d$ on state $\hat{U}\psi$. Since $\hat{\lambda}_j$ is one of Pauli matrices extending to a higher dimension, the corresponding unitary operation $\hat{U}$ is always pretty simple. So we can first perform the unitary operation $\hat{U}$ on state ψ, then measure populations and calculate the expectation value of $\hat{B}_d$. Each population measurement is repeated by 1000 times. For the uncertainty of the measurements, we mainly consider the quantum projection noise and use the standard deviation of projection outcomes. The measurement result of the final physical observable can be always written as a function of expectation values of $\hat{\lambda}_j$ operators. We finally find the error bar by using the standard error propagating method.

Supplementary Note 4: Physical Observables

In this part, we will describe in detail the procedure to extract the information of various physical observables from experimental data. The time-dependent enlarged four-component spinor can be formally written in the momentum space as follows,

$$|\Psi(t)\rangle = \int \Psi(p) |\chi_p(t)\rangle \otimes |p\rangle , \tag{6}$$

with $|p\rangle$ being the momentum basis, i.e. the plane-wave states, and $|\chi_p(t)\rangle$ describing the internal state $|\chi_p(t)\rangle = \sum_{j=1}^{4} \chi_{p,j}(t) |j\rangle$. Note that the wave function in the momentum space $\Psi(p)$ does not depend on time and is fully determined by the initial condition $|\psi(0)\rangle$ in the original space, because the effective Hamiltonian $\hat{\mathcal{H}}$ commutes with the momentum operator $\hat{p}_x$. The only time-dependent part in Eq. (6) is the internal state $|\chi_p(t)\rangle$, whose dynamics is determined by the enlarged space Hamiltonian $\hat{\mathcal{H}}_p = pc(|1\rangle\langle 2| + |3\rangle\langle 4|) + imc^2(|1\rangle\langle 4| - |2\rangle\langle 3|) + \text{H.c.}$. The equation of motion for $|\chi_p(t)\rangle$, given by $i\hbar\partial_t |\chi_p(t)\rangle = \hat{\mathcal{H}}_p |\chi_p(t)\rangle$, can be simulated in a quantum four-level system. Using quantum state tomography, we experimentally obtain the density matrix $\hat{\varrho}_p(t)$ corresponding to $|\chi_p(t)\rangle\langle\chi_p(t)|$.

A. Diagonal operators in the momentum space

The general form of a diagonal operator $\hat{\mathcal{O}}_{\rm dg}$ in the momentum space can be written as follows,

$$\hat{\mathcal{O}}_{\rm dg} = \hat{\Sigma} \otimes f\left(\hat{p}\right), \tag{7}$$

with $\hat{\Sigma} = c_0\hat{\mathbb{I}}+c_1\hat{\sigma}_x+c_2\hat{\sigma}_y+c_3\hat{\sigma}_z$ and $f\left(\cdot\right)$ being an arbitrary algebraic function, $\left\langle p\left|f\left(\hat{p}\right)\right|p'\right\rangle = f\left(p\right)\delta\left(p-p'\right)$. The expectation value of this operator at arbitrary time t can be obtained as follows,

$$\begin{aligned}\left\langle\Psi\left(t\right)\left|M^{\dagger}\hat{\mathcal{O}}_{\rm dg}M\right|\Psi\left(t\right)\right\rangle &= \int dpdp'\Psi^{*}\left(p\right)\Psi\left(p'\right)\left\langle p\left|f\left(\hat{p}\right)\right|p'\right\rangle\left\langle\chi_{p}\left(t\right)\left|M^{\dagger}\hat{\Sigma}M\right|\chi_{p'}\left(t\right)\right\rangle\\ &= \int dp\left|\Psi\left(p\right)\right|^{2}f\left(p\right)\mathrm{Tr}\left[\hat{\varrho}_{p}\left(t\right)\hat{M}^{\dagger}\hat{\Sigma}\hat{M}\right].\end{aligned} \tag{8}$$

We may take the average momentum as a simple example,

$$\begin{aligned}p\left(t\right) &\equiv \left\langle\psi\left(t\right)\left|\hat{p}\right|\psi\left(t\right)\right\rangle \\ &= \left\langle\Psi\left(t\right)\left|M^{\dagger}\hat{p}M\right|\Psi\left(t\right)\right\rangle\\ &= \int dpp\left|\Psi\left(p\right)\right|^{2}\mathrm{Tr}\left[\hat{\varrho}_{p}\left(t\right)\hat{M}^{\dagger}\hat{M}\right].\end{aligned} \tag{9}$$

The quantum simulation for each $\left|\chi_{p}\left(t\right)\right\rangle$ will be as follows.

1. Prepare the initial state $\left|\chi_{p}\left(0\right)\right\rangle = \sum_{j=1}^{4}\chi_{p,j}\left(t\right)\left|j\right\rangle$.

2. Implement the Hamiltonian $\hat{\mathcal{H}}_{p}$ and let the system evolve for certain time duration t,

$$\hat{\mathcal{H}}_{p} = \begin{pmatrix} 0 & cp & 0 & imc^{2}\\ cp & 0 & -imc^{2} & 0\\ 0 & imc^{2} & 0 & cp\\ -imc^{2} & 0 & cp & 0\end{pmatrix}. \tag{10}$$

3. Perform the quantum state tomography and obtain $\hat{\varrho}_{p}\left(t\right) = \left|\chi_{p}\left(t\right)\right\rangle\left\langle\chi_{p}\left(t\right)\right|$.

Then the matrix element mentioned above can be obtained straightforwardly,

$$\left\langle\chi_{p}\left(t\right)\left|M^{\dagger}\hat{\Sigma}M\right|\chi_{p}\left(t\right)\right\rangle = \mathrm{Tr}\left[\hat{\rho}_{p}\left(t\right)M^{\dagger}\hat{\Sigma}M\right]. \tag{11}$$

B. Off-diagonal operators in the momentum space

Then we turn to investigate the method to obtain the expectation value of some off-diagonal operators $\hat{\mathcal{O}}_{\rm od}$ in the momentum space. We will take position-dependent operators as examples, i.e.,

$$\hat{\mathcal{O}}_{\rm od}=\hat{\Sigma}\otimes f\left(\hat{x}\right). \tag{12}$$

As mentioned above, the expectation value can be written as

$$\begin{aligned}&\left\langle \Psi\left(t\right)\left|M^{\dagger}\hat{\mathcal{O}}_{\rm dg}M\right|\Psi\left(t\right)\right\rangle \qquad (13)\\ =&\int dpdp'\Psi^{*}\left(p\right)\Psi\left(p'\right)\left\langle p\left|f\left(\hat{x}\right)\right|p'\right\rangle\left\langle \chi_{p}\left(t\right)\left|M^{\dagger}\hat{\Sigma}M\right|\chi_{p'}\left(t\right)\right\rangle .\end{aligned}$$

Since $f\left(\hat{x}\right)$ is not diagonal in the momentum space, the above expression will involve off-diagonal matrix-element as $\left\langle \chi_{p}\left(t\right)\left|M^{\dagger}\hat{\Sigma}M\right|\chi_{p'}\left(t\right)\right\rangle$. If we stick to the previous scheme, we will obtain two independent density matrices $\hat{\varrho}_{p}\left(t\right)$ and $\hat{\varrho}_{p'}\left(t\right)$, from which we can not construct the off-diagonal matrix element between two distinct momenta.

Inspired by the effective Hamiltonian $\hat{\mathcal{H}}_{p}$ for some definite momentum p,

$$\begin{aligned}\hat{\mathcal{H}}_{p} &= pc\left(|1\rangle\langle 2|+|3\rangle\langle 4|\right)+imc^{2}\left(|1\rangle\langle 4|-|2\rangle\langle 3|\right)+\text{H.c.}\\ &\equiv pc\left(\hat{\mathbb{I}}\otimes\hat{\sigma}_{x}\right)-mc^{2}\left(\hat{\sigma}_{x}\otimes\hat{\sigma}_{z}\right), \qquad (14)\end{aligned}$$

we notice that the first qubit can be diagonalized in the $\hat{\sigma}_{x}$-basis. The quantum states and operators in the new basis $\{|+\rangle|0\rangle, |+\rangle|1\rangle, |-\rangle|0\rangle, |-\rangle|1\rangle\}$ and the old basis $\{|0\rangle|0\rangle, |0\rangle|1\rangle, |1\rangle|0\rangle, |1\rangle|1\rangle\}$, where $|0\rangle$ and $|1\rangle$ are the eigenstates of $\hat{\sigma}_{z}$, are related by the following transform matrix $\hat{S}$,

$$\hat{S}=\frac{\sqrt{2}}{2}\begin{pmatrix}1&0&1&0\\0&1&0&1\\1&0&-1&0\\0&1&0&-1\end{pmatrix}, \tag{15}$$

where $|\pm\rangle\equiv\frac{1}{\sqrt{2}}\left(|0\rangle\pm|1\rangle\right)$ are eigenstates of $\hat{\sigma}_{x}$. In other words, the equation of motion for $|\chi_{p}\left(t\right)\rangle$ can be written in the new basis as follows,

$$i\hbar\frac{\partial}{\partial t}\begin{pmatrix}\chi_{p}^{+}\left(t\right)\\ \chi_{p}^{-}\left(t\right)\end{pmatrix}=\begin{pmatrix}\hat{H}_{p}^{+}&0\\0&\hat{H}_{p}^{-}\end{pmatrix}\begin{pmatrix}\chi_{p}^{+}\left(t\right)\\ \chi_{p}^{-}\left(t\right)\end{pmatrix} \tag{16}$$

with

$$\begin{pmatrix} \hat{H}_p^+ & 0 \\ 0 & \hat{H}_p^- \end{pmatrix} = \hat{S}^\dagger \hat{H}(p)\hat{S} = \begin{pmatrix} 0 & pc+imc^2 & 0 & 0 \\ pc-imc^2 & 0 & 0 & 0 \\ 0 & 0 & 0 & pc-imc^2 \\ 0 & 0 & pc+imc^2 & 0 \end{pmatrix} \tag{17}$$

and

$$\begin{pmatrix} \chi_p^+(t) \\ \chi_p^-(t) \end{pmatrix} = \begin{pmatrix} \chi_{p,1}^+(t) \\ \chi_{p,2}^+(t) \\ \chi_{p,1}^-(t) \\ \chi_{p,2}^-(t) \end{pmatrix} = \hat{S}^\dagger \begin{pmatrix} \chi_{p,1}(t) \\ \chi_{p,2}(t) \\ \chi_{p,3}(t) \\ \chi_{p,4}(t) \end{pmatrix}, \tag{18}$$

where $\chi_p^\pm(t)$ are column vectors with two entries and $\hat{H}_p^\pm$ are 2×2 matrices in the new basis. As shown in Eq. (16), we note that the dynamics for $\chi_p^\pm(t)$ are totally decoupled from each other, and can be separately simulated in quantum two-level systems. In order to obtain off-diagonal matrix elements between two distinct momenta p and p', we have to simulate $\chi_p(t)$ and $\chi_{p'}(t)$ coherently. We obtain the following equations of motion by rearranging Eq. (16),

$$\begin{aligned} i\hbar\frac{\partial}{\partial t}\begin{pmatrix} \chi_p^+(t) \\ \chi_{p'}^+(t) \end{pmatrix} &= \begin{pmatrix} \hat{H}_p^+ & 0 \\ 0 & \hat{H}_{p'}^+ \end{pmatrix}\begin{pmatrix} \chi_p^+(t) \\ \chi_{p'}^+(t) \end{pmatrix}, \\ i\hbar\frac{\partial}{\partial t}\begin{pmatrix} \chi_p^-(t) \\ \chi_{p'}^-(t) \end{pmatrix} &= \begin{pmatrix} \hat{H}_p^- & 0 \\ 0 & \hat{H}_{p'}^- \end{pmatrix}\begin{pmatrix} \chi_p^-(t) \\ \chi_{p'}^-(t) \end{pmatrix}, \end{aligned} \tag{19}$$

which can be simulated in quantum four-level systems.

In the following investigation, we will use the average position $\langle \hat{x}\rangle \equiv \langle \psi(t)|\hat{x}|\psi(t)\rangle$ as an example. The detailed derivation is as follows,

$$\begin{aligned} \langle \hat{x}\rangle &= \left\langle \Psi(t)\left|M^\dagger \hat{x} M\right|\Psi(t)\right\rangle \\ &= \int dpdp'\Psi^*(p)\Psi(p')\langle p|\hat{x}|p'\rangle\left\langle \chi_p(t)\left|M^\dagger M\right|\chi_{p'}(t)\right\rangle \\ &= \int dpdp'\Psi^*(p)\Psi(p') \\ &\quad\times\langle p|\hat{x}|p'\rangle\left[\left\langle \chi_p^+(t)|\chi_{p'}^+(t)\right\rangle + \left\langle \chi_p^-(t)|\chi_{p'}^-(t)\right\rangle - i\left(\left\langle \chi_p^+(t)|\chi_{p'}^-(t)\right\rangle - \left\langle \chi_p^-(t)|\chi_{p'}^+(t)\right\rangle\right)\right] \\ &= \int \frac{dxdpdp'}{2\pi\hbar}x\exp\left[-(p-p')x/\hbar\right]\Psi^*(p)\Psi(p')\left[\left\langle \chi_p^+(t)|\chi_{p'}^+(t)\right\rangle + \left\langle \chi_p^-(t)|\chi_{p'}^-(t)\right\rangle\right]. \end{aligned}$$

The last line in the above equation is valid because of the following identity,

$$\int \frac{dpdp'}{2\pi\hbar} \exp\left[-\left(p-p'\right)x/\hbar\right] \Psi^*\left(p\right)\Psi\left(p'\right)\left[\left\langle \chi_p^+\left(t\right)|\chi_{p'}^-\left(t\right)\right\rangle - \left\langle \chi_p^-\left(t\right)|\chi_{p'}^+\left(t\right)\right\rangle\right] = 0, \quad (20)$$

which can be verified using $\rho_E\left(p\right) = \rho_E\left(-p\right)$ and $\chi_p^{\pm}\left(t\right) = \left[\chi_{-p}^{\pm}\left(t\right)\right]^*$.

The experiment procedure would be as follows.

1. Prepare the initial state determined by the initial condition $\left[\chi_{p,1}^{\pm}\left(0\right), \chi_{p,2}^{\pm}\left(0\right), \chi_{p',1}^{\pm}\left(0\right), \chi_{p',2}^{\pm}\left(0\right)\right]^T$.

2. Implement $\hat{H}_{p,p'}^{\pm}$ and let the system evolve for some time period t,

$$\hat{H}_{p,p'}^{\pm} = \begin{pmatrix} \hat{H}_p^{\pm} & 0 \\ 0 & \hat{H}_{p'}^{\pm} \end{pmatrix} = \begin{pmatrix} 0 & pc \pm imc^2 & 0 & 0 \\ pc \mp imc^2 & 0 & 0 & 0 \\ 0 & 0 & 0 & p' \pm imc^2 \\ 0 & 0 & p' \mp imc^2 & 0 \end{pmatrix}. \quad (21)$$

3. Perform the quantum state tomography and obtain $\rho_{p,p'}^{\pm}$,

$$\rho_{p,p'}^{\pm} = \begin{pmatrix} \left|\chi_{p,1}^{\pm}\right|^2 & \chi_{p,1}^{\pm}\left(\chi_{p,2}^{\pm}\right)^* & \chi_{p,1}^{\pm}\left(\chi_{p',1}^{\pm}\right)^* & \chi_{p,1}^{\pm}\left(\chi_{p',2}^{\pm}\right)^* \\ \chi_{p,2}^{\pm}\left(\chi_{p,1}^{\pm}\right)^* & \left|\chi_{p,2}^{\pm}\right|^2 & \chi_{p,2}^{\pm}\left(\chi_{p',1}^{\pm}\right)^* & \chi_{p,2}^{\pm}\left(\chi_{p',2}^{\pm}\right)^* \\ \chi_{p',1}^{\pm}\left(\chi_{p,1}^{\pm}\right)^* & \chi_{p',1}^{\pm}\left(\chi_{p,2}^{\pm}\right)^* & \left|\chi_{p',1}^{\pm}\right|^2 & \chi_{p',1}^{\pm}\left(\chi_{p',2}^{\pm}\right)^* \\ \chi_{p',2}^{\pm}\left(\chi_{p,1}^{\pm}\right)^* & \chi_{p',2}^{\pm}\left(\chi_{p,2}^{\pm}\right)^* & \chi_{p',2}^{\pm}\left(\chi_{p',1}^{\pm}\right)^* & \left|\chi_{p',2}^{\pm}\right|^2 \end{pmatrix}. \quad (22)$$

Sweeping the momenta p and p' over all possible values, we would obtain all of the information that is needed to calculate the expectation value $x\left(t\right) \equiv \left\langle \psi\left(t\right)|\hat{x}|\psi\left(t\right)\right\rangle$. The number of separate simulations for different $\left(p,p'\right)$ pairs for both signs will be N_P^2, where N_P is the number of points with which we discretize the momentum axis.

Supplementary Note 5: Charge conservation and charge conjugation

The non-Hermitian Majorana Hamiltonian does not have eigenstates. However, we can define the concepts of particle and antiparticle from the eigenstates of the corresponding Dirac Hamiltonian, which is obtained by substituting the Majorana mass term with the Dirac mass term. Under the same convention, the $1+1$ Dirac equation takes the following dimensionless form,

$$i\partial_t\left|\psi\right\rangle = \left(\hat{\sigma}_x\hat{p}_x + m\hat{\sigma}_z\right)\left|\psi\right\rangle, \quad (23)$$

with the eigenvalues $\pm\sqrt{p^2+m^2}$ and the corresponding eigenstates

$$\left|\psi_p^{(+)}\right\rangle = \frac{1}{\sqrt{2}\left(p^2+m^2\right)^{1/4}}\begin{pmatrix}\sqrt{\sqrt{p^2+m^2}+m}\\ \left(p/\left|p\right|\right)\sqrt{\sqrt{p^2+m^2}-m}\end{pmatrix}\otimes\left|p\right\rangle, \tag{24}$$

$$\left|\psi_p^{(-)}\right\rangle = \frac{1}{\sqrt{2}\left(p^2+m^2\right)^{1/4}}\begin{pmatrix}\sqrt{\sqrt{p^2+m^2}-m}\\ -\left(p/\left|p\right|\right)\sqrt{\sqrt{p^2+m^2}+m}\end{pmatrix}\otimes\left|p\right\rangle.$$

Starting from an initial Majorana spinor $\left|\psi\left(0\right)\right\rangle = \binom{u_1(0)}{u_2(0)}\otimes\left|p\right\rangle$, the time-dependent Majorana spinor can be formally written as follows,

$$\left|\psi\left(t\right)\right\rangle = \begin{pmatrix}v_1\left(t\right)\\ v_2\left(t\right)\end{pmatrix}\otimes\left|-p\right\rangle + \begin{pmatrix}u_1\left(t\right)\\ u_2\left(t\right)\end{pmatrix}\otimes\left|p\right\rangle. \tag{25}$$

Note that the appearance of the negative momentum component is originated from the charge conjugation in the Majorana mass term. By definition, the time-dependent charge is obtained as follows,

$$C\left(t\right) = \left|\left\langle\psi_p^{(+)}|\psi\left(t\right)\right\rangle\right|^2 + \left|\left\langle\psi_{-p}^{(+)}|\psi\left(t\right)\right\rangle\right|^2 - \left|\left\langle\psi_p^{(-)}|\psi\left(t\right)\right\rangle\right|^2 - \left|\left\langle\psi_{-p}^{(-)}|\psi\left(t\right)\right\rangle\right|^2. \tag{26}$$

By setting $\binom{u_1(0)}{u_2(0)} = \binom{1}{0}$, we obtain the theoretical and experimental data shown in Fig. 2 (b) in the main text.

Besides the violation of the charge conservation of plane-wave initial states, we investigate the charge conjugation on top of the Majorana dynamics of an initial moving Gaussian wave packet as shown in Figs. 3 **e-h** in the main text. The initial Majorana spinor takes form of $\psi\left(x,t=0\right) = \frac{1}{2}\pi^{-1/4}e^{-x^2/8-ip_0x}\binom{1}{1}$ with $p_0 = 1$ in the position space. By definition, the charge conjugation interchanges the particle and antiparticle components in Eq. (25). In addition to the results in the main text, here we show the theoretical result for the dynamics of the internal degree of freedom in Fig. 1. In Fig. 1 **B**, we can clearly see that the populations of the particle and antiparticle components are interchanged right after the implementation of the charge-conjugation operator. Fig. 1 **C** shows the momentum distributions for the particle and antiparticle components at different times above and below the base lines, respectively. We clearly see from the Majorana dynamics of the internal degree of freedom that the evolution is continued after the implementation of the charge conjugation, although the roles of the particle and antiparticle are interchanged.

Supplementary Note 6: Microwave Implementation

In our $^{171}Yb^{+}$ ion system, we use the microwaves for the transitions between $|1\rangle$ and $|2\rangle, |3\rangle, |4\rangle$ levels. We use a microwave Raman scheme similar to the widely used Raman laser scheme for the transitions between $|2\rangle$ to $|3\rangle$ and $|3\rangle$ to $|4\rangle$ transitions. We cannot apply a radio frequency for the operations of these transitions, since the energy gap between $|2\rangle \leftrightarrow |3\rangle$ and $|3\rangle \leftrightarrow |4\rangle$ is very close, which is $(2\pi)31$ kHz for our experimental condition. As shown in Fig. 1 of the main text, 6 different frequencies of microwaves are combined and simultaneously applied to the trap. For the control of 6 microwaves, we use a PCI-board arbitrary waveform generator (AWG) with 1 GHz sampling rate, which is mixed with a 12442.8213 MHz microwave. The AWG generates the signal of 6 frequencies from 186 $\sim$ 214 MHz.

The system is described by the Hamiltonian $\hat{H} = \hat{H}_A + \hat{H}_{AL}$, with atomic part $\hat{H}_A$ being

$$\hat{H}_A = \left(\hbar\omega_{\text{hf}} - \hbar\omega_z\right)|2\rangle\langle 2| + \left(\hbar\omega_{\text{hf}} + \omega_q\right)|3\rangle\langle 3| + \left(\hbar\omega_{\text{hf}} + \hbar\omega_z\right)|4\rangle\langle 4|, \tag{27}$$

and the interaction part $\hat{H}_{AL}$ being

$$\hat{H}_{AL}(t) = \sum_{n=1}^{6}\sum_{j=2}^{4} \hbar\Omega_{1j}^{(n)} \cos\left(\omega_n t + \phi_n\right)\hat{\sigma}_x^{(j)}, \tag{28}$$

respectively. We set the 6 frequencies in the microwave as follows,

$$\begin{aligned}
\omega_1 &= \omega_{\text{hf}} - \omega_z - \delta_1, & \omega_2 &= \omega_{\text{hf}} + \omega_z - \delta_2, \\
\omega_3 &= \omega_{\text{hf}} - \omega_z - \Delta, & \omega_4 &= \omega_{\text{hf}} + \omega_q - \Delta - \delta_3, \\
\omega_5 &= \omega_{\text{hf}} + \omega_q + \Delta, & \omega_6 &= \omega_{\text{hf}} + \omega_z + \Delta - \delta_4,
\end{aligned} \tag{29}$$

where Δ is the detuning for the stimulated Raman transitions and δ_i are the frequency shifts used to compensate the AC Stark effect. Using the method in Ref. [1], we obtain the effective Hamiltonian $\hat{\mathcal{H}}_{\text{eff}} = \hat{\mathcal{H}}_{\text{st}} + \hat{\mathcal{H}}_{\text{cp}}$ in the interaction picture defined by $\hat{H}_A$, where $\hat{\mathcal{H}}_{\text{st}}$

includes all of the AC Stark shifts,

$$\begin{aligned}\hat{\mathcal{H}}_{\rm st} \;=\;& \frac{\hbar\left(\Omega_{13}^{(1)}\right)^2}{4\left(\omega_z+\omega_q+\delta_1\right)}\left(|3\rangle\langle 3|-|1\rangle\langle 1|\right)+\frac{\hbar\left(\Omega_{14}^{(1)}\right)^2}{4\left(2\omega_z+\delta_1\right)}\left(|4\rangle\langle 4|-|1\rangle\langle 1|\right)\\
&-\frac{\hbar\left(\Omega_{12}^{(2)}\right)^2}{4\left(2\omega_z-\delta_2\right)}\left(|2\rangle\langle 2|-|1\rangle\langle 1|\right)-\frac{\hbar\left(\Omega_{13}^{(2)}\right)^2}{4\left(\omega_z-\omega_q-\delta_2\right)}\left(|3\rangle\langle 3|-|1\rangle\langle 1|\right)\\
&+\frac{\hbar\left(\Omega_{12}^{(3)}\right)^2}{4\Delta}\left(|2\rangle\langle 2|-|1\rangle\langle 1|\right)+\frac{\hbar\left(\Omega_{13}^{(3)}\right)^2}{4\left(\omega_z+\Delta+\omega_q\right)}\left(|3\rangle\langle 3|-|1\rangle\langle 1|\right)\\
&+\frac{\hbar\left(\Omega_{14}^{(3)}\right)^2}{4\left(2\omega_z+\Delta\right)}\left(|4\rangle\langle 4|-|1\rangle\langle 1|\right)\\
&-\frac{\hbar\left(\Omega_{12}^{(4)}\right)^2}{4\left(\omega_z-\Delta+\omega_q-\delta_3\right)}\left(|2\rangle\langle 2|-|1\rangle\langle 1|\right)+\frac{\hbar\left(\Omega_{13}^{(4)}\right)^2}{4\left(\Delta+\delta_3\right)}\left(|3\rangle\langle 3|-|1\rangle\langle 1|\right)\\
&+\frac{\hbar\left(\Omega_{14}^{(4)}\right)^2}{4\left(\omega_z+\Delta-\omega_q+\delta_3\right)}\left(|4\rangle\langle 4|-|1\rangle\langle 1|\right)\\
&-\frac{\hbar\left(\Omega_{12}^{(5)}\right)^2}{4\left(\omega_z+\Delta+\omega_q\right)}\left(|2\rangle\langle 2|-|1\rangle\langle 1|\right)-\frac{\hbar\left(\Omega_{13}^{(5)}\right)^2}{4\Delta}\left(|3\rangle\langle 3|-|1\rangle\langle 1|\right)\\
&+\frac{\hbar\left(\Omega_{14}^{(5)}\right)^2}{4\left(\omega_z-\Delta-\omega_q\right)}\left(|4\rangle\langle 4|-|1\rangle\langle 1|\right)\\
&-\frac{\hbar\left(\Omega_{12}^{(6)}\right)^2}{4\left(\omega_z+\Delta-\delta_4\right)}\left(|2\rangle\langle 2|-|1\rangle\langle 1|\right)-\frac{\hbar\left(\Omega_{13}^{(6)}\right)^2}{4\left(\omega_z+\Delta-\omega_q-\delta_4\right)}\left(|3\rangle\langle 3|-|1\rangle\langle 1|\right)\\
&-\frac{\hbar\left(\Omega_{14}^{(6)}\right)^2}{4\left(\Delta-\delta_4\right)}\left(|4\rangle\langle 4|-|1\rangle\langle 1|\right)\\
\;=\;& \hbar\omega_{\rm st}^{(1)}|1\rangle\langle 1|+\hbar\omega_{\rm st}^{(2)}|2\rangle\langle 2|+\hbar\omega_{\rm st}^{(3)}|3\rangle\langle 3|+\hbar\omega_{\rm st}^{(4)}|4\rangle\langle 4|, \end{aligned} \tag{30}$$

and $\hat{\mathcal{H}}_{\rm cp}$ includes all coupling terms with frequencies much smaller than Δ,

$$\begin{aligned}\hat{\mathcal{H}}_{\rm cp}=&\frac{\hbar\widetilde{\Omega}_{12}e^{-i\delta_1 t+i\phi_1}}{2}|1\rangle\langle 2|+\frac{\hbar\widetilde{\Omega}_{14}e^{-i\delta_2 t+i\phi_2}}{2}|1\rangle\langle 4| \\
&+\frac{\hbar\widetilde{\Omega}_{23}e^{-i\delta_3 t+i\phi_{43}}}{2}|2\rangle\langle 3|+\frac{\hbar\widetilde{\Omega}_{34}e^{-i\delta_4 t+i\phi_{65}}}{2}|3\rangle\langle 4|\\
&+\frac{\hbar\Omega_{13}^{(3)}\Omega_{14}^{(4)}\left(\omega_z+\Delta\right)}{4\left[\left(\omega_z+\Delta\right)^2-\omega_q^2\right]}|3\rangle\langle 4|e^{i(2\omega_q-\delta_3)t+i\phi_{43}}-\frac{\hbar\Omega_{12}^{(5)}\Omega_{13}^{(6)}\left(\omega_z+\Delta\right)}{4\left[\left(\omega_z+\Delta\right)^2-\omega_q^2\right]}|2\rangle\langle 3|e^{-i(2\omega_q+\delta_4)t+i\phi_{65}}+\text{H.c.}\end{aligned} \tag{31}$$

The effective couplings $\widetilde{\Omega}_{23}$ and $\widetilde{\Omega}_{34}$ are defined as follows,

$$\widetilde{\Omega}_{23}=\frac{\Omega_{12}^{(3)}\Omega_{13}^{(4)}}{4}\left(\frac{1}{\Delta}+\frac{1}{\Delta+\delta_3}\right),\quad \widetilde{\Omega}_{34}=-\frac{\Omega_{13}^{(5)}\Omega_{14}^{(6)}}{4}\left(\frac{1}{\Delta}+\frac{1}{\Delta-\delta_4}\right). \tag{32}$$

Transferring into the second rotating frame defined by $\hat{\mathcal{H}}_{\rm st}$, one obtains the following rotating frame Hamiltonian

$$\hat{\mathcal{H}} = e^{i\hat{\mathcal{H}}_{\rm st}t/\hbar}\hat{\mathcal{H}}_{\rm cp}\left(t\right)e^{-i\hbar\mathcal{H}_{\rm st}t/\hbar}. \tag{33}$$

In order to make the final rotating frame Hamiltonian time-independent, the additional detunings should satisfy the following relations,

$$\begin{aligned} \delta_1 &= \omega_{\rm st}^{(1)} - \omega_{\rm st}^{(2)}, \\ \delta_2 &= \omega_{\rm st}^{(1)} - \omega_{\rm st}^{(4)}, \\ \delta_3 &= \omega_{\rm st}^{(2)} - \omega_{\rm st}^{(3)}, \\ \delta_4 &= \omega_{\rm st}^{(3)} - \omega_{\rm st}^{(4)}. \end{aligned} \tag{34}$$

Comparing to the Majorana equation, one obtains the following relations

$$\begin{aligned} \frac{\hbar\widetilde{\Omega}_{12}}{2}e^{i\phi_1} &= \frac{\hbar\widetilde{\Omega}_{34}}{2}e^{i\phi_{65}} = cp, \\ \frac{\hbar\widetilde{\Omega}_{14}}{2}e^{i\phi_2} &= -\frac{\hbar\widetilde{\Omega}_{23}}{2}e^{i\phi_{43}} = imc^2. \end{aligned} \tag{35}$$

One problem in this scheme is the slowing down of operations. The Raman transition is 10 times slower than normal Rabi flopping, even with full power. But now we need 6 microwaves together. Decoherence occurs when the whole microwave duration is longer than 600μs. This decoherence problem is later solved by applying a line trigger [2] to the pulse sequencer.

Supplementary References

---

[1] D. F. V. James and J. Jerke, arXiv:quant-ph/0706.1090 (2007).

[2] A. Smith, B. E. Anderson, S. Chaudhury, P. S. Jessen, J. Phys. B: At. Mol. Opt. Phys. **44**, 205002 (2011).

[3] R. T. Thew, K. Nemoto, A. G. White, W. J. Munro, Phys. Rev. A **66** 012303 (2002).

[4] R. A. Bertlmann, P. Krammer, J. Phys. A: Math. Theor. **41** 235303 (2008).
